# Systematic minimization of RNA ligase ribozyme through large-scale design-synthesis-sequence cycles

**DOI:** 10.1093/nar/gkz729

**Published:** 2019-08-26

**Authors:** Yoko Nomura, Yohei Yokobayashi

**Affiliations:** Nucleic Acid Chemistry and Engineering Unit, Okinawa Institute of Science and Technology Graduate University, Onna, Okinawa 904 0495, Japan

## Abstract

Template-directed RNA ligation catalyzed by an RNA enzyme (ribozyme) is a plausible and important reaction that could have been involved in transferring genetic information during prebiotic evolution. Laboratory evolution experiments have yielded several classes of ligase ribozymes, but their minimal sequence requirements remain largely unexplored. Because selection experiments strongly favor highly active sequences, less active but smaller catalytic motifs may have been overlooked in these experiments. We used large-scale DNA synthesis and high-throughput ribozyme assay enabled by deep sequencing to systematically minimize a previously laboratory-evolved ligase ribozyme. After designing and evaluating >10 000 sequences, we identified catalytic cores as small as 18 contiguous bases that catalyze template-directed regiospecific RNA ligation. The fact that such a short sequence can catalyze this critical reaction suggests that similarly simple or even simpler motifs may populate the RNA sequence space which could have been accessible to the prebiotic ribozymes.

## INTRODUCTION

Although no direct molecular signatures exist, it is widely accepted that RNA preceded DNA and proteins in the hypothetical RNA world (1,2) in which RNA assumed the roles of storing and transferring genetic information as well as catalyzing essential biochemical reactions to sustain life. Template-directed RNA ligation between a 3′-OH end of an RNA fragment and a 5′ triphosphorylated RNA fragment (Supplementary Figure S1A) is an important reaction in the RNA world, as it represents information transfer mediated by RNA using the same chemistry found in the DNA and RNA polymerase proteins in modern organisms. However, since no RNA enzymes (ribozymes) that catalyze this reaction have survived natural evolution, researchers have relied on laboratory evolution to isolate and characterize such ribozymes.

One RNA ligase ribozyme (class I Bartel ligase) that was discovered through *in vitro* selection from a large pool of random RNA sequences can catalyze template-directed RNA ligation very efficiently (*k*_obs_ > 1 s^−1^) (3). This rather large (∼180 nt) and structurally complex ligase was further engineered to function as RNA polymerase ribozymes through a series of laboratory evolution experiments (4–8). However, it is unlikely that such a sophisticated ribozyme could have emerged by chance during the earliest phase of prebiotic evolution. Slower but smaller ligase ribozymes are more likely to have emerged first, eventually forming self-replicating catalytic networks as experimentally demonstrated by the Joyce group (9,10). Consequently, elucidation of minimal sequence requirements for RNA ligase ribozyme activity has implications for the probability of emergence of prebiotic catalytic RNAs.

There are several classes of small ligase ribozymes that catalyze 3′-5′ ligation reaction between 3′-OH and 5′-triphosphate termini in a template-directed manner. Ikawa and coworkers combined 3D molecular modeling and *in vitro* selection to design the DSL (Supplementary Figure S1B) and YFL ribozymes with well-defined catalytic and substrate recognition domains (11,12). Robertson and Ellignton discovered the L1 ligase from random RNA sequences (13). The L1 ligase has been extensively engineered and characterized, and the 3D structure of a minimized variant (14) in its product form was solved (15). The minimized L1 ligase contained a catalytic core (excluding substrate binding arms) of ∼35 nt (Supplementary Figure S1C) showing that relatively small RNA sequences are capable of catalyzing RNA ligation.

**Figure 1.**
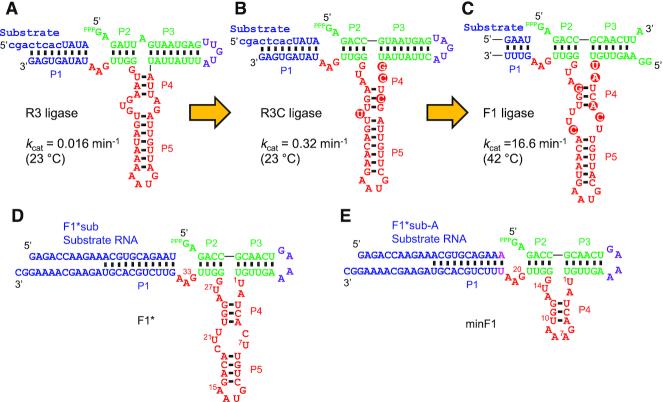
Precursors and derivatives of the F1 ligase ribozyme. (A–C) Stepwise evolution of the fast F1 ligase from the ancestral R3 ligase by the Joyce group. Major base changes in the catalytic core are highlighted. (**A**) R3 ligase isolated by *in vitro* selection from random RNA sequences lacking cytidine (16). Lowercase letters in the substrate indicate DNA bases. (**B**) R3C ligase isolated after partial randomization of R3 ligase with all four bases and *in vitro* selection (16). (**C**) The catalytic core of the F1 ligase (17) that was selected for higher catalytic and self-replication efficiency from E1 (10) whose catalytic core is essentially identical to that of R3C. The substrate and the substrate-binding region of the ligase are abbreviated. (**D**) F1* ligase based on the F1 ligase by Robertson and Joyce (17). (**E**) A truncated variant of F1* (minF1) designed based on the large-scale mutational analysis. Green: triphosphorylated ‘substrate’ and substrate-binding regions of the ribozyme, purple: terminal loop, red: catalytic core of the ribozyme subjected to mutations, blue: external substrate and substrate binding region of the ribozyme.

Another ribozyme that appears to fold into a three-way junction structure similar to the L1 ligase was discovered by Rogers and Joyce from a random pool of RNA sequences that totally lack cytidine (16). This ribozyme, R3, was further allowed to use cytidine through *in vitro* selection to improve the catalytic rate by 20-fold (R3C) (16). R3C was later optimized for higher self-replication efficiency to yield the F1 ligase with a *k*_cat_ of 16.6 min^−1^ which is more than an order of magnitude faster than L1 and DSL ligases (17). Although both F1 and L1 ligases are predicted to fold into similar secondary structures, no sequence similarities exist at the primary sequence level. Even within the same lineage of ribozymes, R3, R3C and F1 display high variability within the presumed catalytic core (Figure 1A–C).

These discoveries of multiple distinct classes of RNA ligase ribozymes (big and small) pose an intriguing question about the probability of the emergence of RNA ligase activity. One path to address this question is to define minimal sequence requirements of the ribozymes. Smaller ribozymes with fewer sequence constraints are more likely to have emerged during prebiotic evolution, therefore, lend more support to the RNA world even if they are less catalytically active. However, as the previous laboratory evolution efforts of these ribozymes focused on optimizing catalytic activity through *in vitro* selection and sequence analysis, they have likely overlooked many weakly active sequences that were outcompeted during selection experiments. Few efforts to minimize these ribozymes have relied on *ad hoc* examination of several variants based on intuitive trimming of terminal stem-loops (18).

In this work, we performed two cycles of high-throughput assay of F1-based ribozyme variants based on deep sequencing to elucidate the minimal sequence requirements of the ribozyme. We identified key sequence requirements and base-base interactions in the F1-like ribozyme that were previously not reported, resulting in a modified secondary structure model of the ribozyme core. We also found that a catalytic core as small as 18 bases can catalyze the reaction at a moderate rate, which represents the smallest catalytic core for this class of ribozymes. Our systematic large-scale mutational analysis of a ribozyme is free from selection bias, therefore, should help advance our understanding of ribozyme sequence-function relationships.

## MATERIALS AND METHODS

### Preparation of ligase ribozyme libraries

DNA templates that encode the T7 promoter and ribozyme variants were synthesized as oligo pools (CustomArray Inc.) with the following sequence: 5′ CCTAATACGACTCACTATA-[ribozyme sequence] 3′ (T7 promoter underlined). The oligo pools were amplified by PCR using primers Ligase-lib-f and Ligase-lib-r (Supplementary Table S1) using Phusion High-Fidelity PCR Master Mix with HF Buffer (NEB). The PCR products were purified by silica columns (DNA Clean & Concentrator-5, Zymo Research) and used as templates for *in vitro* transcription using HiScribe T7 High Yield RNA Synthesis Kit (NEB) or ScriptMAX Thermo T7 Transcription Kit (Toyobo) according the manufacturers’ instructions. Upon completion of the transcription reaction, an equal volume of DNase I (NEB) stock solution (1:1:3 mixture of 2 U/μl DNase I, 10 × DNase I Reaction Buffer, and nuclease-free water) was added and incubated for 10 min at 37°C. RNA was cleaned by a silica column (RNA Clean & Concentrator-5, Zymo Research) and eluted in nuclease-free water. The ligase ribozyme pools were further purified by denaturing (TBE-urea) polyacrylamide gel electrophoresis (PAGE). The gels were stained by SYBR Gold (Thermo Fisher) and visualized on a blue-light transilluminator. The bands of the expected sizes were excised and frozen at −80°C, crushed, and the RNAs were extracted in Tris/NaCl buffer (30 mM Tris–HCl, pH 7.5, 30 mM NaCl) at 4°C for 4 h. The RNAs were precipitated by ethanol using Quick-Precip Plus Solution (EdgeBio), washed twice with 70% ethanol, and resuspended in nuclease-free water.

### Ligation reactions of ribozyme libraries

A ligase ribozyme pool (0.67 μM) was mixed with the appropriate substrate (F1*sub or F1*subA) at 6.7 μM in nuclease-free water. The RNA solution was heated to 72°C for 3 min and then cooled on ice. Prior to the start of the ligation reaction, the RNA solution and the 4 × reaction buffer (200 mM EPPS pH 8.5, 100 mM MgCl_2_, 8 U/μl RNase Inhibitor, Murine (NEB)) were separately incubated at 42°C for at least 2 min. The reaction was initiated by mixing 3 volumes of RNA solution and 1 volume of 4 × reaction buffer, followed by 30 min incubation at 42°C. The reaction was terminated by adding 2.25 volumes of cold stop solution (5:13 mixture of 0.5 M EDTA and RNA Loading Dye (2×) (NEB)) and kept on ice. The F1* library ligation was performed in 8 μl and the minF1 library in 24 μl.

### Preparation of sequencing templates

The sequencing library construction process is illustrated in Supplementary Figure S2A. The quenched reaction solutions were heated to 95°C for 3 min and cooled on ice, and the samples were separated on a TBE–urea 8% polyacrylamide gel. The bands corresponding to the ligated and unligated ribozymes were excised and separately extracted as described above. The ligated and unligated RNAs were dissolved in an equal volume of nuclease-free water (10 μl for F1* library and 21 μl for minF1 library) and 5 μl was used for reverse transcription reactions. Reverse transcription was performed in 10 μl scale using Maxima H Minus Reverse Transcriptase (Thermo Fisher) according to the manufacturer's instructions using R1-504+2nt-F1*lig or R1-503-F1*lig (Supplementary Table S1) as a primer for the unligated and ligated fractions, respectively. The reverse transcription reaction was allowed to proceed for 30 min at 65°C and the enzyme was inactivated at 85°C for 5 min. Subsequently, RNAs were digested by adding 1/20 volume of 5 N NaOH and incubating at 95°C for 3 min. The cDNAs were then purified by denaturing PAGE as described above to remove the unreacted primers. The excised gel fragments from unligated and ligated cDNAs were combined at this stage and cDNAs were coextracted as described above for RNA extraction and resuspended in 10 μl nuclease-free water. Primers R2-F1*lig and R1-f (Supplementary Table S1) were used in PCR to amplify the cDNA mixture using Phusion High-Fidelity PCR Master Mix with HF Buffer. The PCR product was diluted and used as the template in a second PCR using primers Adapter-T2-new and B2-CGAGTAAT (Supplementary Table S1). The final PCR products (Supplementary Figure S2B) were purified by agarose gel electrophoresis using Zymoclean Gel DNA Recovery Kit (Zymo Research). The DNA concentration was measured by real-time PCR (StepOnePlus, Thermo Fisher) using NEBNext Library Quant Kit for Illumina (NEB), and analyzed using MiSeq Reagent Kit v3 with 15% PhiX Control v3 (Illumina).

### Sequencing data analysis

MiSeq sequence data (fastq files) were analyzed by custom Python scripts. For the F1* library, each raw sequence read was first quality filtered for having at least 70% of the base calls with quality core (QS) ≥ 20. After trimming the adapter sequence and the constant ribozyme sequence, the catalytic core sequence was further quality filtered to have all base calls with QS ≥ 20. The sequence reads that passed the quality check were sorted to either ligated or unligated pools based on the 8 or 10 nt barcode sequence embedded in the reverse transcription primers. The number of reads of each ligase variant were then counted in the ligated (*N*_lig_) and the unligated (*N*_unlig_) pools which were used to calculate the fraction ligated (FL) values. The minF1 library was analyzed similarly except that the adapter sequence and the constant ribozyme sequence was first removed from each raw sequence read, and then the remaining catalytic core sequence was quality filtered to have all base calls with QS ≥ 30.

### PAGE analysis of individual ligase ribozymes

DNA templates encoding the T7 promoter followed by individual ribozyme sequences 5′ CCTAATACGACTCACTATA-[ribozyme sequence] 3′ (T7 promoter underlined) were prepared by annealing and extending two synthetic oligonucleotides using OneTaq 2X Master Mix with Standard Buffer (NEB). The dsDNA templates were then purified by silica columns (DNA Clean & Concentrator-5) and were used for *in vitro* transcription reactions as described above. Ligation reactions were performed as described above except for using the ligase ribozyme in excess (2 μM) over the FAM-labeled substrate (FAM-F1*subA, 0.1 μM, FASMAC). Polyacrylamide gels were imaged using Typhoon FLA9500 (GE Healthcare) and quantified with ImageJ software. Kinetic analyses of minF1 and 4d394 were performed similarly by sampling 4 μl of the reaction mixture at appropriate time-points and mixing it with 9 μl cold stop solution. FAM-F1*sub was used as the substrate for the kinetic analysis of F1*.

### Deoxyribozyme cleavage of ligation products

minF1 and 4d394 were ligated with F1*subA and the ligations products were purified by denaturing PAGE as described above. As controls, the expected ligation products were prepared by *in vitro* transcription using corresponding DNA templates prepared by annealing and extending two oligonucleotides as described above. The RNA (12.5–27.6 ng) was mixed with 3.5 μM Dz8-17 (Supplementary Table S1) in 4 μl of the Dz annealing buffer (5 mM Tris–HCl pH 7.5, 15 mM NaCl, 0.1 mM EDTA) and incubated at 95°C for 3 min. After cooling on ice for 5 min, deoxyribozyme reaction was initiated by adding 1 μl of the Dz reaction buffer (175 mM Tris–HCl pH 7.5, 675 mM NaCl, 300 mM MgCl_2_, 3.64 U/μl RNase Inhibitor, Murine). The cleavage was allowed to proceed for 90 min at 37°C. Dz8-17 was digested by adding 5 μl of TURBO DNase (Thermo Fisher) stock solution (1:1:3 mixture of 2 U/μl TURBO DNase, 10× TURBO DNase Buffer, and nuclease-free water) and incubation at 37°C for 10 min. The reaction products were separated by denaturing PAGE as described above, stained with SYBR Gold, and imaged by LuminoGraph II (ATTO).

## RESULTS

The catalytic core of F1* derived from the F1 ribozyme of Robertson and Joyce (17) includes 35 bases (U1-A35) as shown in Figure 1D. U1-G28 constitute the main core sequence that includes stems P4, P5, two internal loops, and one terminal loop. G29-U32 forms the P2 stem acting as a spacer to position the ligation site opposite to the GAA (positions 33–35) bulge.

Previous efforts to elucidate the sequence-function relationships of nucleic acid enzymes have relied on either statistical randomization of a parental sequence by doped oligonucleotide synthesis or error-prone PCR, or saturation mutagenesis of a defined set of positions via oligonucleotide synthesized containing degenerate bases (14,17). While these strategies, coupled with appropriate selection methods and sequencing of the functional (selected) variants, yield some sequence–function relationship information, the accessible sequence space is mostly limited to base substitutions. In the case of statistical mutagenesis, the population is also strongly biased for the mutants with fewer mutations. The applied selection pressure also determines the arbitrary threshold of catalytic activity which is difficult to control. Biases due to PCR can also affect the selected population. It has been pointed out that the number of any particular mutant within a population does not necessarily reflect the intrinsic activity (19), and it is only a qualitative indication that the mutant possesses a certain level of activity.

**Figure 2.**
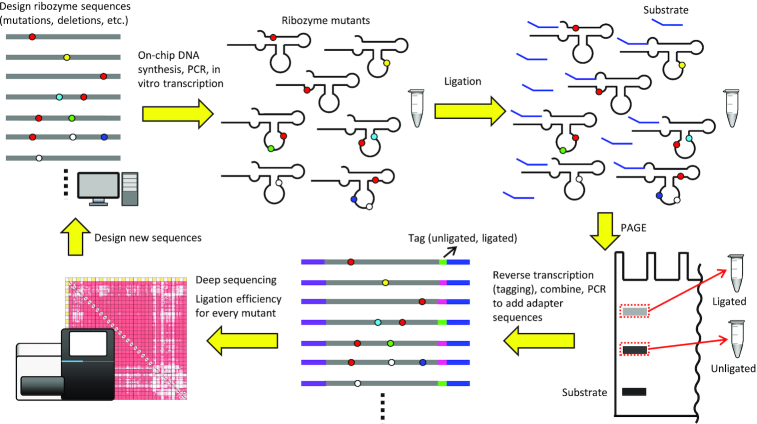
Outline of the ribozyme library preparation, deep sequencing analysis, and sequence design processes.

To gain the global sequence-function relationship of F1*, we designed 6967 variants of the ribozyme that include the original sequence (WT: wild-type), all single and double mutants, and all single, double, and triple deletions in the main catalytic core and the GAA bulge (positions 1–28, 33–35 in Figure 1D). Eighty-one additional mutants not included in these sequences were also designed. Such an arbitrary set of mutants cannot be synthesized efficiently by statistical mutagenesis or degenerate oligonucleotides. Therefore, we utilized commercial on-chip parallel synthesis of the desired sequences provided as an oligo pool. The oligo pool was used to construct the *in vitro* transcription template by PCR. Then the ribozyme mutants were obtained as a mixture after *in vitro* transcription by T7 RNA polymerase (Figure 2).

The ribozymes were allowed to ligate with an excess amount of the substrate F1*sub (Figure 1D) for 30 min at 42°C. The reaction mixture was then separated on a denaturing polyacrylamide gel, and the ligated and unligated ribozyme bands were separately excised. RNAs extracted from each band were separately reverse-transcribed with a primer containing a barcode sequence to identify the source (ligated or unligated). The cDNAs were gel-purified, mixed, and amplified by PCR to attach adapter sequences for Illumina sequencing (Figure 2). The library was sequenced by MiSeq and the data were analyzed by counting the number of reads for every mutant with both ligated (*N*_lig_) and unligated (*N*_unlig_) barcodes. Fraction ligated (FL) was calculated as:

$$\begin{equation*}{\rm{FL}} = {N_{{\rm{lig}}}}/\left( {{N_{{\rm{lig}}}} + {N_{{\rm{unlig}}}}} \right)\end{equation*}$$

Relative activity (RA) of each mutant was calculated by dividing FL by that of the wild-type (0.826). It should be noted that the FL and RA values represent ribozyme activity measured at a single time-point (30 min). Due to the high *k*_obs_ of the parental ribozyme, RA cannot differentiate variants with *k*_obs_ greater than ∼0.1 min^−1^ (see Discussion). Therefore, the RA should be interpreted as a semi-quantitative parameter with a limited dynamic range especially at high *k*_obs_.

**Figure 3.**
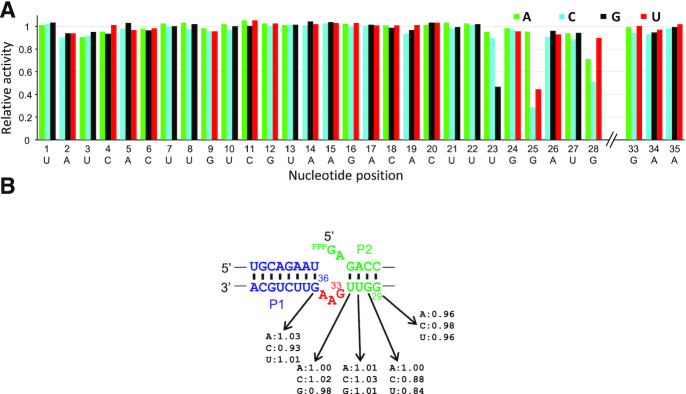
Relative activities of the single mutants of F1* in (**A**) the catalytic core and (**B**) near the ligation junction.

Most of the 93 single mutants were highly active (85/93 with RA>0.90). Only three substitutions (U23G, G25C, G25U) resulted in RA falling below 0.50 (Figure 3A). The least active mutant (G25C, RA = 0.29) still retained appreciable activity, suggesting that the ribozyme is highly tolerant to mutations. In contrast to most natural ribozymes, there are no functionally critical bases that render the ribozyme inactive by a single mutation.

Tolerance of the ribozyme to mutations was further confirmed by the analysis of 4185 double mutants (Supplementary Figure S3, Supplementary Data 1). Approximately 66% of the mutants showed RA > 0.90. However, 6.1% of the mutants showed RA < 0.10 indicating that cumulative negative effects of multiple mutations emerge in some double mutants. The 2D plot of the double mutants reveal that the sensitivity of the ribozyme activity to mutations varies within the ribozyme sequence. Double mutations within U23-A35 are particularly sensitive. Some mutations within this region (U23G, G25C, G25U, G28A, G28C) that showed weaker activity as single mutants (Figure 3A) result in generally lower activity in combination with other mutations throughout the ribozyme. Another interesting observation is that the ribozyme is extremely tolerant to double mutations within A5-U22.

Single base deletions generally do not affect the ribozyme activity (Supplementary Figure S4A). Only A26del and U27del resulted in significantly lower RAs, 0.43 and 0.20, respectively. Naturally, many of the double deletions that include either A26del or U23del resulted in loss of activity. Double deletions within A5-U22 essentially maintained the WT activity, consistent with the double substitutions within this region (Supplementary Figure S4B). Triple deletion data also highlight the robustness of the ribozyme sequence, with 37% (846/2283) of the mutants displaying RA > 0.90 (Supplementary Data 1).

Flexibility of on-chip oligonucleotide synthesis was exploited by including a series of arbitrarily designed sequences. We focused on the GAA bulge (positions 33–35) opposite to the ligation site, to test all possible bulge sequences with sizes 0–3 (Table 1). All single mutants within the bulge are highly active, as are most of the double mutants. A conspicuous exception is ucA (mutations in lowercase) which is essentially inactive. A possible explanation is that the newly introduced UC sequence hybridizes with the 5′GA of the ribozyme to extend the P2 stem. In fact, all UCN (N = A/C/G/U) bulges were inactive. Most of the bulge triple mutants were also inactive.

**Table 1.** Relative activities of F1* mutants in the G33-A35 bulge

Bulge sequence	RA	Bulge sequence	RA	Bulge sequence	RA	Bulge sequence	RA
GAA (WT)	1.00	3-nt bulge, double mutants		3-nt bulge, triple mutants		2-nt bulge	
3-nt bulge, single mutants		acA	0.93	acc	0.06	AA	1.07
aAA	1.05	agA	1.05	acg	0.10	AC	0.71
cAA	0.99	auA	1.02	acu	0.19	AG	0.99
uAA	1.06	ccA	0.28	agc	0.17	AU	0.97
GcA	0.98	cgA	1.08	agg	0.43	CA	1.01
GgA	1.00	cuA	0.94	agu	0.19	CC	0.03
GuA	1.03	ucA	0.02	auc	0.02	CG	0.03
GAc	1.04	ugA	1.03	aug	0.18	CU	0.02
GAg	1.05	uuA	0.48	auu	0.21	GA	1.08
GAu	1.08	aAc	1.00	ccc	0.03	GC	1.02
		aAg	1.06	ccg	0.03	GG	0.45
		aAu	1.02	ccu	0.04	GU	0.45
		cAc	0.53	cgc	0.13	UA	1.07
		cAg	0.99	cgg	0.06	UC	0.01
		cAu	0.81	cgu	0.03	UG	0.03
		uAc	0.92	cuc	0.01	UU	0.02
		uAg	1.01	cug	0.04	1-nt bulge	
		uAu	1.01	cuu	0.02	A	0.99
		Gcc	0.57	ucc	0.00	C	0.02
		Gcg	0.03	ucg	0.01	G	0.03
		Gcu	0.98	ucu	0.01	U	0.02
		Ggc	0.96	ugc	0.08	No bulge	
		Ggg	1.03	ugg	0.05	-	0.03
		Ggu	0.49	ugu	0.03		
		Guc	0.10	uuc	0.01		
		Gug	0.28	uug	0.01		
		Guu	0.58	uuu	0.02		

For the 3-nt bulge mutants, the mutations are indicated in lowercase.

There is a clear pattern in the 2-nt bulge variants. AN, NA and GN are at least partially active (RA>0.45) while all other mutants were inactive. Furthermore, A is the only active single-bulge mutant, and deletion of the bulge altogether renders the ribozyme inactive. During the laboratory evolution of F1, Robertson and Joyce discovered GA and AA bulge variants after 10 rounds of *in vitro* evolution (17) which is consistent with our observation. However, no other active bulge variants were observed probably due to the stringent selection pressure employed and the low sequence coverage by Sanger sequencing.

In another set of mutants, the length of P2 (4 bp) was reduced to 1–3 bp resulting in change in the relative distance between the main catalytic core and the ligation site. Partial activity was observed when P2 was reduced to 3 bp (s35, RA = 0.29) while shorter P2 (s36, s37) completely inactivated the ribozyme (Supplementary Table S2). All single mutations in P2 between the main catalytic core and the GAA bulge (positions 29–32) were also examined. Surprisingly, all mutants were highly active (RA > 0.84) even those that bear a mismatch in P2 closest to the ligation site. Similarly, mutations at G36 which forms a G-U pair with the 3′ end of the substrate were also tolerated (Supplementary Table S2, s31–s33, RA > 0.92). These results indicate surprising mutational tolerance near the ligation site (Figure 3B).

Overall, the large-scale mutational analysis of F1* points to its high tolerance to mutations (including base deletions). This is consistent with the extensive sequence variations observed during the evolution of the ancestral R3 ribozyme which lacks cytidines to R3C, and subsequently to F1 by the Joyce group (16,17). Apart from the overall secondary structure, the GAA bulge, and A26-G28, there are no strictly conserved sequence motifs. Furthermore, our results indicate that even these seemingly conserved nucleotides are not functionally essential. Because selection favors higher activity, conservation of sequence motifs after selection does not always mean that they are functionally essential. Whether a certain sequence motif is functionally essential can only be established by assaying appropriate mutants.

**Figure 4.**
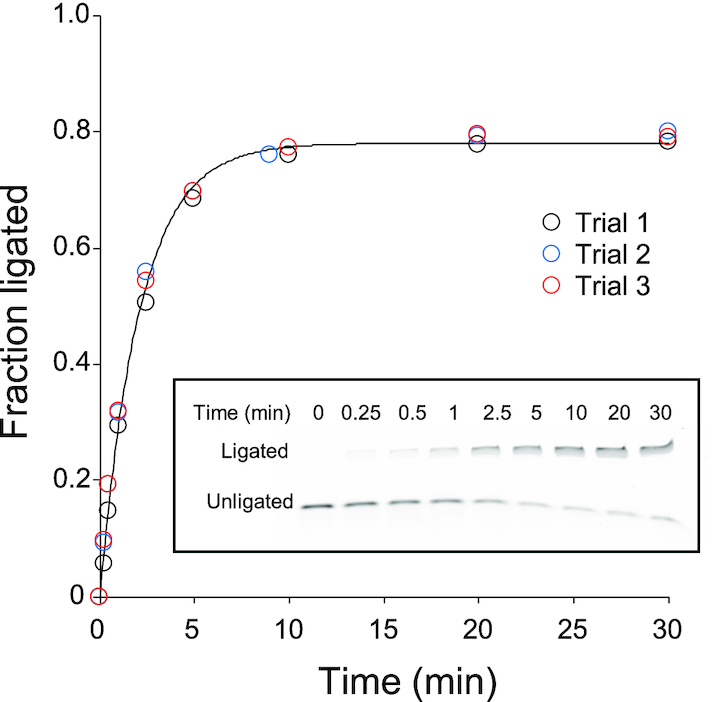
Ligation reaction of minF1 and FAM-F1*subA (FAM: 6-fluorescein label). minF1 was in excess and the samples were analyzed by denaturing PAGE. FAM fluorescence was detected by a gel imager. The experiment was repeated three times, and the data were fitted to the equation FL = *F*_max_(1 – e^−^*^k^*_obs_^t^) which is represented by the solid curve. A representative gel electrophoregram is shown in inset.

Based on these observations, we set out to discover a minimal set of nucleotides in F1* that retains the RNA ligase function. Considering the conspicuous robustness of the ribozyme to double mutations and double deletions in A5-U22 and the secondary structure of F1/F1* proposed by Joyce and coworkers, we designed minF1 by replacing C7-U22 of F1* with a short GAAA tetraloop (Figure 1E). Another small modification was made at the 3′ end of the substrate and the corresponding base in minF1: the 3′ end of the substrate was changed to from U to A, and the complementary position 23 in minF1 was changed to U. This was done to allow analysis of the regiospecificity at the ligation junction (A-G) by deoxyribozyme 8–17 (Dz8-17) which specifically cleaves the native 3′-5′ linkage between 5′-ApG-3′ but not a 2′-5′ linkage (20,21). We found minF1 to be highly active (*k*_obs_ = 0.48 min^−1^) (Figure 4), confirming that the bases in C7-U22 in F1* are not functionally essential. It should be noted, however, that minF1 is at least one order of magnitude slower compared to F1* which was too fast to be measured at 42°C. Based on the reactions performed at 4°C and 15°C (Supplementary Figure S5), *k*_obs_ of F1* at 42°C was estimated to be 7.0 min^−1^ which is in reasonable agreement with the catalytic rate constant (*k*_cat_) reported for F1 (16.6 min^−1^) using a rapid-quench device under the same reaction conditions (17). It should also be noted that the *k*_obs_ values reported in this work are based on the rate measurements under a single reaction condition in which the ribozyme is present in excess over the substrate, therefore, are only approximations of the true *k*_cat_ values.

We further characterized minF1 by performing another round of large-scale mutation/deletion analysis by sequencing (Supplementary Data 2). The parental minF1 showed FL = 0.76 which was used to normalize the activities of other variants. First, minF1 and 13 variants that exhibit a range of FL values were individually synthesized and assayed for their ligase activities by conventional PAGE analysis (Supplementary Figure S6). The FL values derived from sequencing data showed an excellent correlation with those based on PAGE experiments (*R*^2^ = 0.98), therefore, the RA values obtained by sequencing are highly reliable.

**Figure 5.**
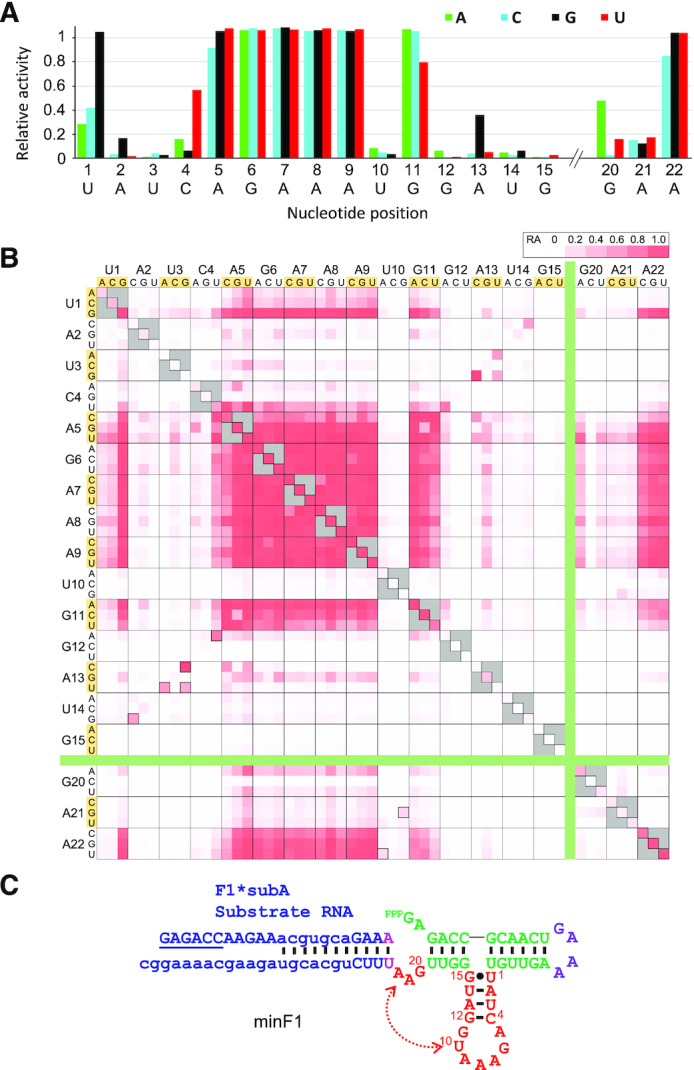
Mutational analysis of minF1. Relative activities of the single (**A**) and double (**B**) mutants of minF1 in the catalytic core. (**C**) A modified secondary structure model of minF1 based on the large-scale mutational analysis

In contrast to the F1* mutants, the single mutants of minF1 include a number of critical bases in the catalytic core. For example, any mutations at U3, U10, G12, U14 or G15 greatly reduce ribozyme activity (RA < 0.10). On the other hand, A5-A9, G11 and A22 are highly tolerant to base substitutions (Figure 5A). A closer inspection of the double mutants reveals additional insights into the sequence-function relationship of the compact ribozyme (Figure 5B). Mutations A2C and U14G both inactivate the ribozyme as single mutants, but the activity is partially restored as a double mutant (RA = 0.58). Similar compensatory effects of mutations U3A/A13U and U3G/A13C, and high activity of the C4U/G12A double mutant suggest Watson-Crick or wobble base-pairs between positions 2–4 and 12–14, leading us to alter the putative secondary structure of the main catalytic core as shown in Figure 5C. This structure is consistent with the secondary structure of the ligated product predicted by the *RNAfold* web server (Supplementary Figure S7) (22). Double mutations within A5-G11 in the loop in the modified secondary structure are well tolerated, with the exception of U10. All single and double mutants that include U10 are essentially inactive. However, U10G/A21G (RA = 0.26) and U10A/A22U (RA = 0.21) are weakly active which may indicate a tertiary interaction between U10 and the GAA bulge.

The bulge mutants of sizes 0–3 bases in minF1 display more stringent sequence requirements compared to F1* in which many bulge variants are tolerated. Only the GAN bulges (RA > 0.80) were highly active in 3-nt bulges, and AAA showing a moderate activity (RA = 0.48). The only other active (RA > 0.50) variant was the 2-nt bugle GA (RA = 0.84) (Supplementary Table S3).

**Figure 6.**
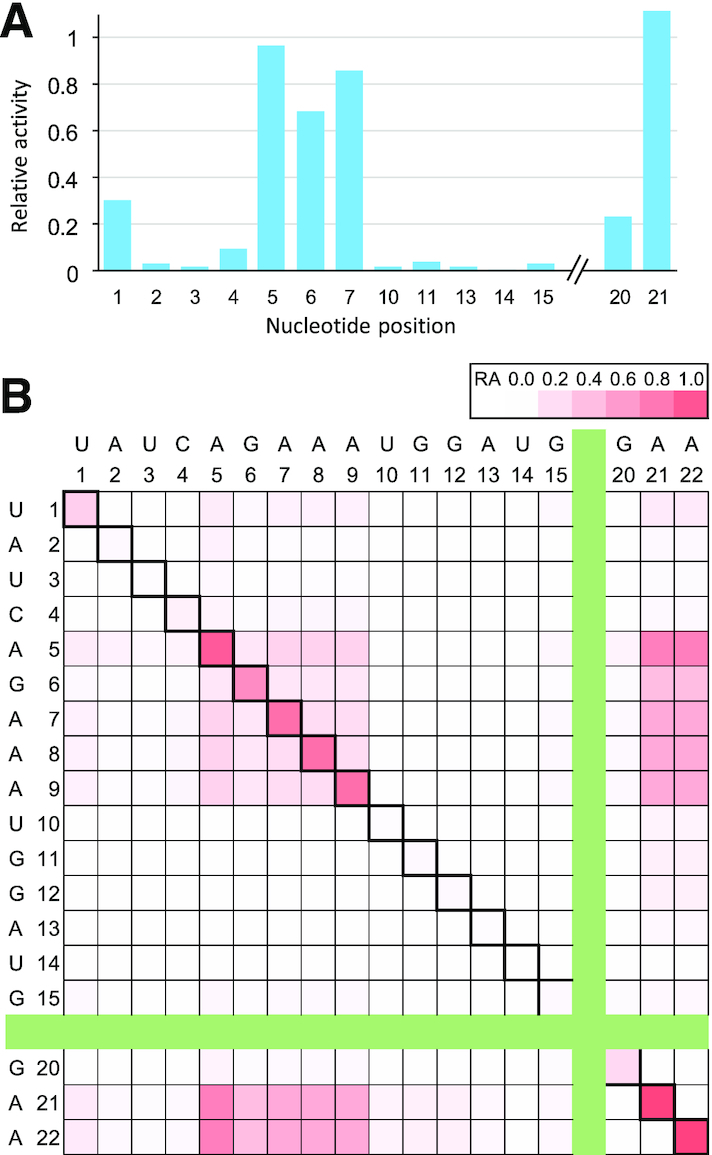
Relative activities of deletion mutants of minF1 in the catalytic core. (**A**) Single base deletions. Note that deletions at some positions (e.g. A8) are abbreviated due to duplications of the resulting mutants. (**B**) Double deletions. The diagonal cells represent single deletion mutants.

minF1 also showed a higher sensitivity to base deletions compared to F1* (Figure 6). Single base deletions in A5–A9 and A21 were well tolerated (RA > 0.68), but all other single base deletions were highly disruptive. U1del (RA = 0.30) and G20del (RA = 0.23) showed some residual activity (Figure 6A). Double deletions within A5–A9 were mostly detrimental (RA < 0.30), but some double deletion mutants in A5–A9 and A21 were moderately active (RA = 0.40–0.76) (Figure 6B).

**Figure 7.**
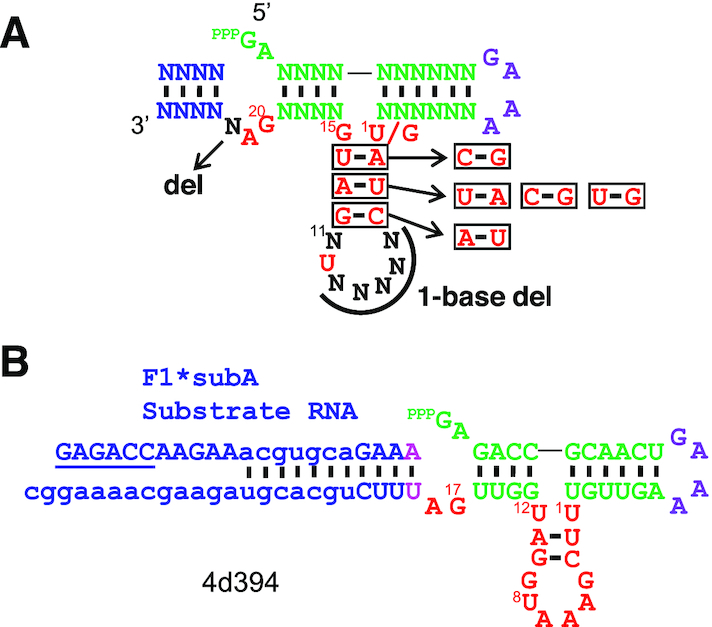
Mutational analysis of minF1. (**A**) Summary of tolerated mutations (base substitutions and deletions) in minF1. (**B**) Structure of 4d394, a 4-base deletion mutant of minF1 with a detectable activity (RA = 0.33).

Overall, the global mutational landscape of minF1 reveals key sequence and structural requirements of the ribozyme better than the larger (and more active) F1*. The above results were visually summarized in Figure 7A. Deletions of 3 and 4 bases resulted in almost universal inactivation with few exceptions (Supplementary Data 2). Among them, the variants that displayed moderate activities were 4-base deletion mutants 4d394 (A2del/A5del/G15del/A21del) and 4d208 (U1del/A5del/G15del/A21del) both showing RA = 0.33 (Figure 7B). It is notable that these mutants are more active than any of the 3-base deletion mutants. In fact, the deletions A2del and G15del abolish activity (RA < 0.04) as single base deletions. Therefore, multi-base deletions can, in some cases, result in synergistic and positive effects even when individual deletions are detrimental. 4d394 was kinetically analyzed over a 7-hour period. Curve fitting of the observed results yielded *k*_obs_ = 0.49 h^−1^ which is ∼58-fold slower compared to minF1 (Supplementary Figure S8). Nevertheless, it is remarkable that a catalytic core as small as 18-nt can catalyze template-directed RNA ligation. Rohatgi *et al.* investigated nonenzymatic, template-directed ligation of RNA fragments with the same ligation chemistry (23). Their results suggest a *k*_obs_ of approximately 3.6 × 10^−5^ h^−1^ for a reaction in 25 mM Mg^2+^, pH 8.9, at 37°C which is four orders of magnitude slower than 4d394.

Regiospecificity of minF1 and 4d394 were confirmed by cleaving the ligation products with deoxyribozyme 8–17 (Dz8-17) targeting the ligation junction. Dz8-17 specifically cleaves 3′-5′ linkage at GA junction but not 2′-5′ (24). The ligation products of minF1 and 4d394 with F1*subA were purified by PAGE. As controls, the expected ligation products were synthesized by *in vitro* transcription. The ligation products and the corresponding controls were digested by excess Dz8-17, and the digestion products were analyzed by PAGE (Supplementary Figure S9). The digestion efficiencies of the ligation products were comparable to those of the control RNAs, indicating that the ligation occurs via 3′-5′ linkage like the parental F1 ribozyme.

## DISCUSSION

Template-directed RNA ligation of a 3′-OH and a 5′-triphosphorylated RNA fragments is of fundamental interest because the same chemistry is used by the modern DNA and RNA polymerases. However, as nonenzymatic polymerization (templated or nontemplated) of ribonucleotide triphosphates (NTPs) has not been reported, 5′-triphosphorylated RNA fragments may not have been available prior to the emergence of more complex RNA polymerase ribozymes. A separate ribozyme that triphosphorylates 5′-OH RNA fragments may have existed, as demonstrated by the laboratory-evolved ribozymes from the Müller group that use trimetaphosphate as a substrate (25,26). Although their triphosphorylation ribozymes are rather complex, simpler and smaller ribozymes may be waiting to be discovered. Alternatively, primitive RNA ligases may have utilized RNA fragments with other activation chemistry. Templated and nontemplated oligomerization of 5′-phosphorimidazolide (Imp) and 5′-phosphoro(2-methyl)imidazolide (2-MeImp) activated nucleotides have been reported (27) which could have provided 5′-activated oligomeric RNA substrates. Indeed, Ikawa and coworkers have reported adaptation of their YFL ligase ribozyme to accept β-nicotinamide mononucleotide (β-NMN) and adenosine monophosphate (AMP) as the leaving groups in the 5′-activated substrates (12). Consequently, it is conceivable that small ribozymes similar to those studied here may be able to catalyze ligation of RNA fragments with different leaving groups.

*In vitro* selection of statistically mutated ribozyme variants generated by doped oligonucleotide synthesis or error-prone PCR followed by sequence analysis of the selected (active) mutants has traditionally been used to investigate the sequence constraints of ribozymes (14,17). More recently, high-throughput sequencing of ribozyme populations selected by activity (select-and-sequence) has provided more comprehensive overview of the sequence-function landscapes of several ribozymes (28–31). By analyzing conserved or covarying bases among the selected mutants, one can infer functionally critical bases and base-base interactions. However, the results are strongly influenced by the stringency of selection which can also be biased due to factors other than ribozyme activity (e.g. PCR efficiency). As selection results in enrichment of more active mutants, the selected population may be dominated by few highly active mutants while masking slower but still active mutants. It is also difficult or impossible to comprehensively analyze deletion mutants due to the difficulty in preparing libraries. Consequently, systematic minimization of ribozymes (while retaining detectable but not necessarily high activity) has not been well addressed experimentally.

Our group has recently demonstrated the use of high-throughput sequencing to quantitatively assay 10^3^–10^4^ self-cleaving ribozyme and deoxyribozyme variants generated by statistical mutagenesis (using doped oligonucleotides) or local randomization (using degenerate oligonucleotides) (32–37). In the present work, we applied a similar approach to an RNA ligase ribozyme using on-chip custom DNA synthesis that enables pooled synthesis of up to 10^5^ or more arbitrary sequences. This allowed us to not only synthesize all possible single and double mutants within the catalytic core, but also synthesize a comprehensive set of deletion mutants and other arbitrarily designed variants. Furthermore, each variant is represented roughly at a similar frequency in the chip-synthesized libraries whereas statistically mutated libraries are highly biased for variants with fewer mutations.

An intriguing observation was the remarkable robustness of the F1* ligase to base substitutions and deletions (Figure 3, Supplementary Figures S3, S4). This may in part be due to the unique evolutionary history of the F1 ligase which originated from a ribozyme selected without cytidine. Moreover, F1 (and thus F1*) was extensively optimized for speed with a *k*_obs_ >10 min^−1^. However, our sequencing-based ribozyme activity assay was performed at a single time-point of 30 min after reaction initiation. This implies that even a *k*_obs_ as low as 0.1 min^−1^ would be sufficient for the majority (95%) of the ligase to have reacted with the substrate. In other words, the apparent mutational tolerance does not mean that mutations do not affect the absolute *k*_obs_ values. Data from additional (earlier) time-points would be necessary to quantitatively analyze the effects of mutations, as demonstrated previously for a deoxyribozyme (32) and a ribozyme (38).

On the other hand, minF1 was found to be much less tolerant to mutations (Figures 5, 6) compared to F1*. This observation can be explained by the lower *k*_obs_ of minF1 (0.48 min^−1^) which makes a smaller drop in *k*_obs_ to be noticeable as a lower fraction of ligated ribozyme at 30 min. It can also be expected that the removal of P5 in F1* structurally destabilizes minF1, possibly resulting in increased sensitivity to mutations. We also identified few mutants with 4 additional base deletions displaying moderate activities. The functional minF1 variants and the deletion mutants represent compact ribozyme motifs defined by a catalytic core that is as small as 18 contiguous bases (Figure 7B) which is significantly smaller than the previously reported template-directed RNA ligase ribozymes. The fact that such a small motif can catalyze the native 3′-5′ ligation reaction suggests that similarly simple or even simpler motifs may populate the RNA sequence space which could have been readily accessed during the early phase of the RNA world. Mutschler *et al.* recently observed that random 20-mer RNA pools with and without activation chemistry display innate ability to form longer sequences through ligation or recombination in eutectic ice phases over a longer time scale (∼months) (39). Slower but smaller catalytic RNAs deserve further exploration as models of primitive ribozymes.

## Supplementary Material

gkz729_Supplemental_Files
