# Supplementary material for: Systematic minimization of RNA ligase ribozyme through large-scale design-synthesis-sequence cycles
Source: Nucleic Acids Res. 2019 Aug 26;47(17):8950–60. doi: 10.1093/nar/gkz729 (PMC6755084; doi:10.1093/nar/gkz729)
Supplement: gkz729_Supplemental_Files [file gkz729_supplemental_files.zip › Nomura_supplementary_data_rev.pdf]

Supplementary Information

Systematic minimization of RNA ligase ribozyme through large-scale design-synthesis-sequence cycles

Yoko Nomura and Yohei Yokobayashi*

Nucleic Acid Chemistry and Engineering Unit, Okinawa Institute of Science and Technology Graduate University, Onna, Okinawa, 904 0495, Japan

*Correspondence to: yohei.yokobayashi@oist.jp

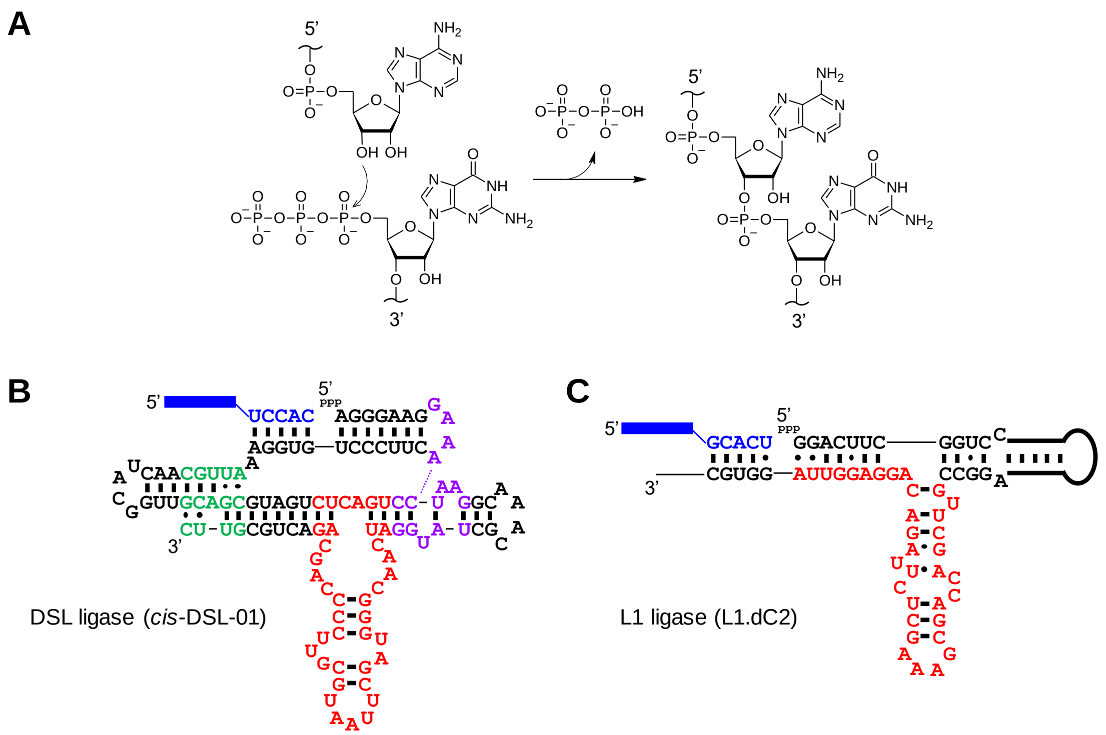

Figure S1. Small template directed RNA ligases that catalyze 3'-5' RNA ligation between 3'-OH and 5'-triphosphorylated termini. (**A**) Mechanism of the ligation reaction. (**B**) DSL ligase by Ikawa et al. (1) (**C**) A truncated variant of the L1 RNA ligase from the Ellington group (2).

A

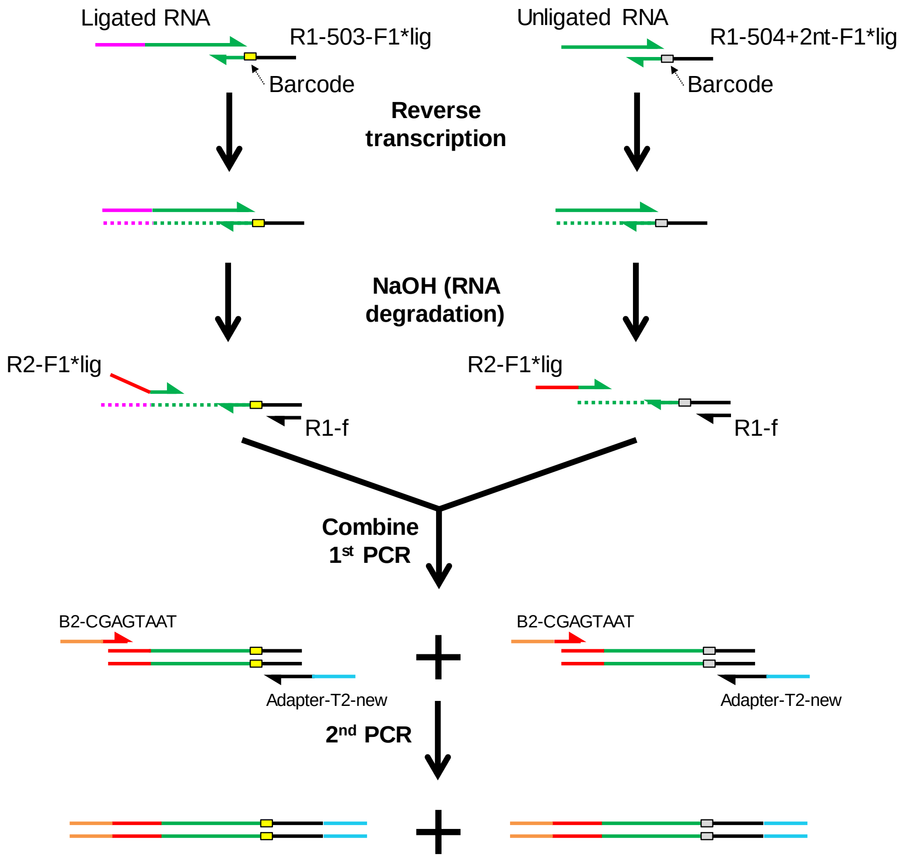

B

Library sequence (5' to 3')

CAAGCAGAAGACGGCATACGAGAT**cgagtaat**GTGACTGGAGTTCAGACGTGTGCTCTTCCGATCT**GAGACCGCAACTG**
AAAAGTTGT[catalytic_core]TTCTGCACGTAGAAGCAAAAGGC[barcode]AGATCGGAAGAGCGTCGTGTAG
GGAAAGAGTGTGTGTAGATCTCGGTGGTCGCCGTATCATT

P7-**index**-**R2**-**[ligase]-barcode**-**R1**-P5

Barcode: AGAGGATA (ligated), TGTCTACTCT (unligated)

Figure S2. Sequencing library construction. (**A**) Schematic illustration of the library construction process. (**B**) Design of the sequencing library. P5 and P7 are the adapter sequences for cluster formation. The index sequence was not used. R1 and R2 are sequencing primer annealing sites (only R1 was used). Barcode indicates if the original ribozyme was ligated or unligated.

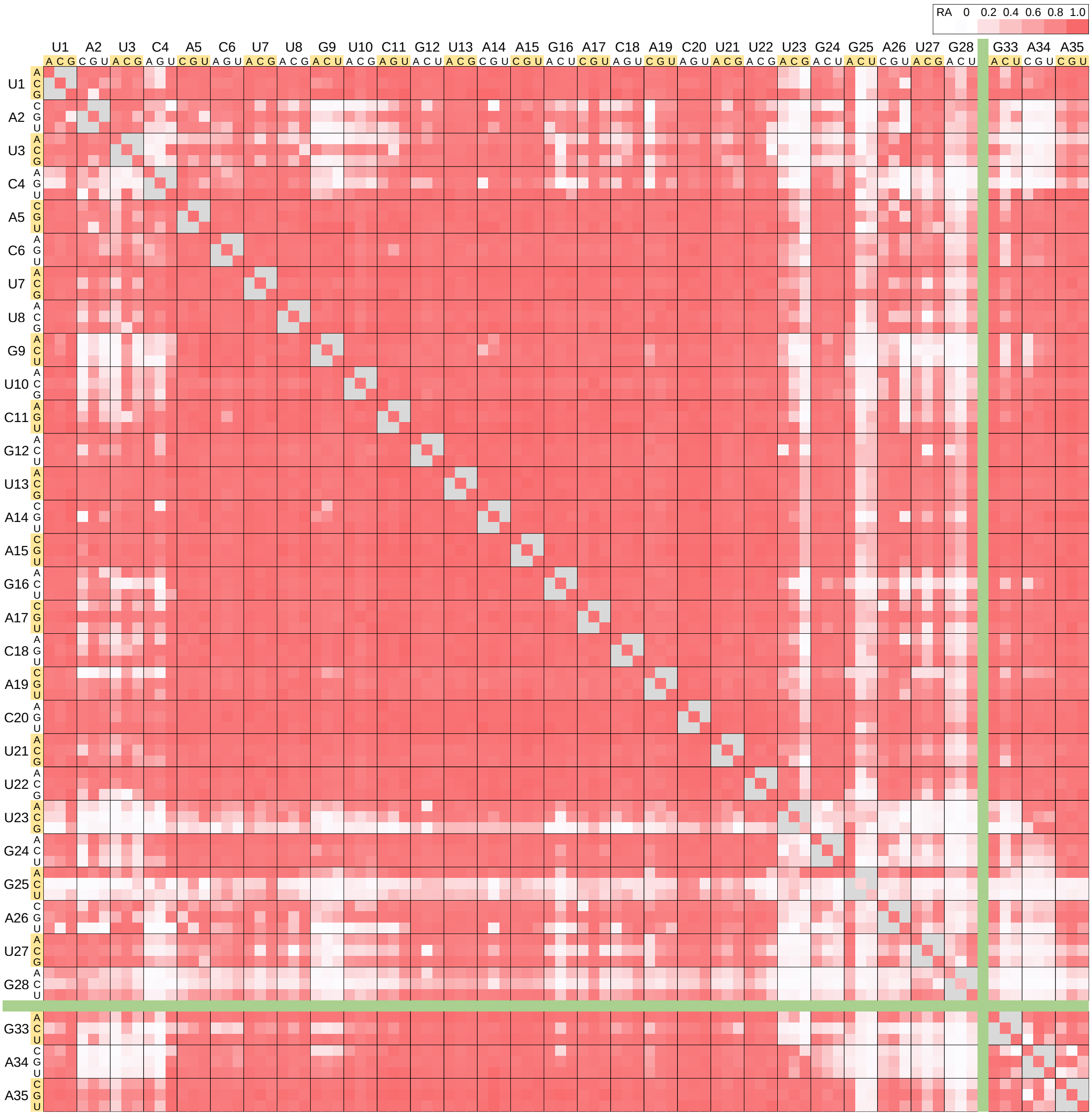

Figure S3. Relative activities of the double mutants of F1* in the catalytic core. The diagonal cells represent single mutants.

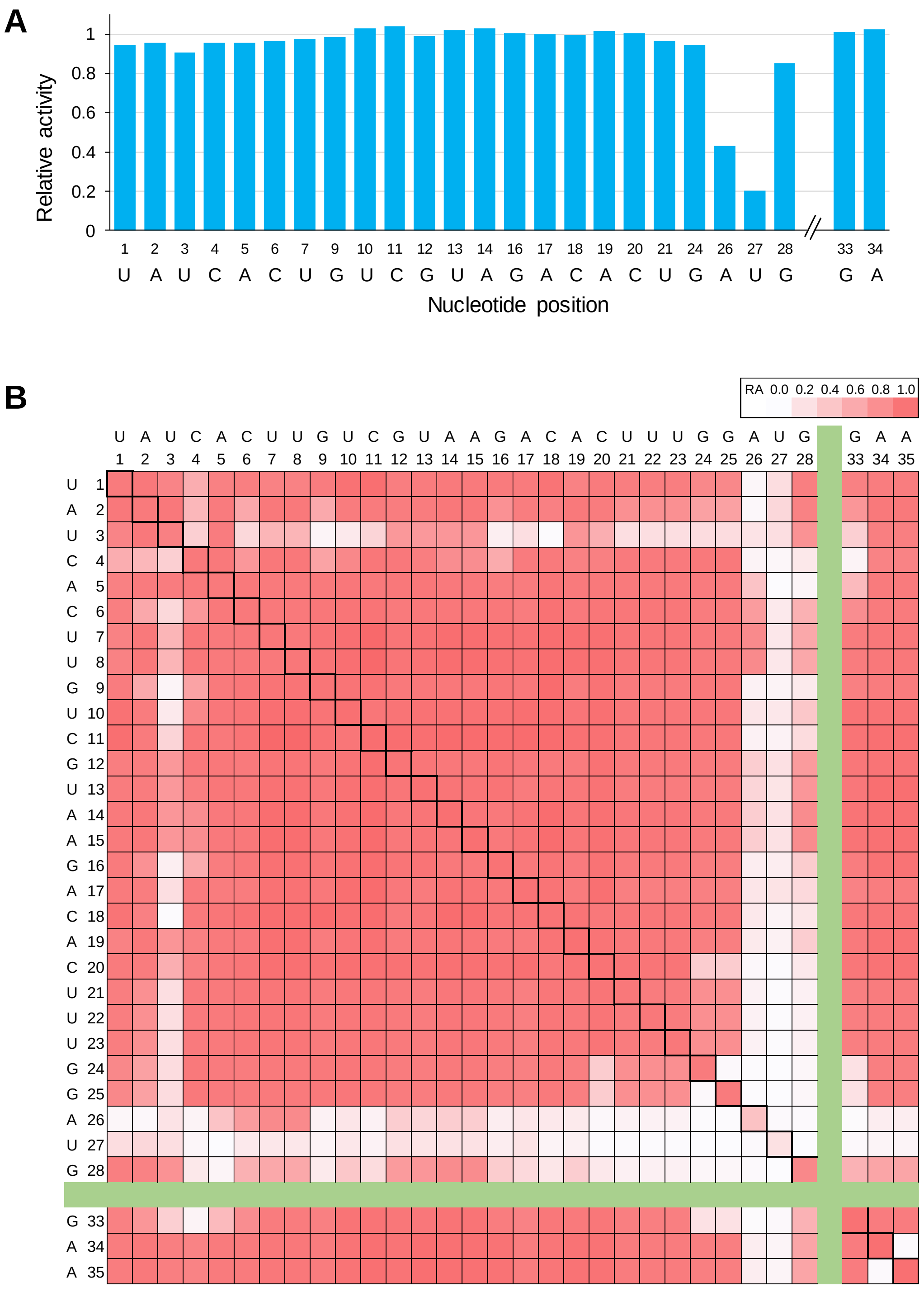

Figure S4. Relative activities of deletion mutants of F1* in the catalytic core. (**A**) Single base deletions. Note that deletions at some positions (e.g. U8) are abbreviated due to duplications of the resulting mutants. (**B**) Double deletions. The diagonal cells represent single deletion mutants.

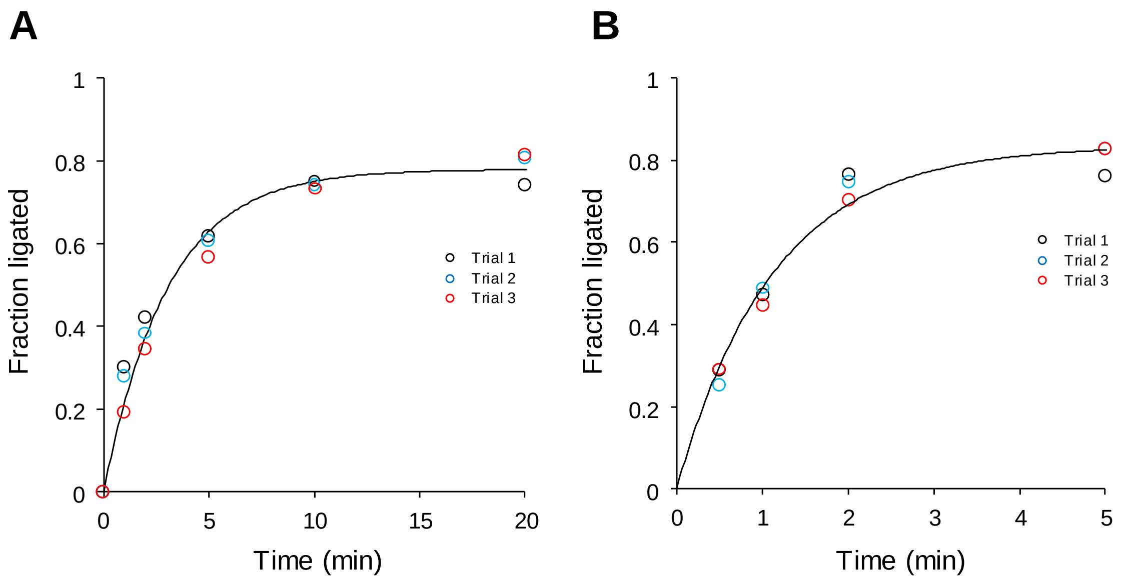

Figure S5. Ligation reactions of F1* and FAM-F1*sub at (**A**) 4 °C and (**B**) 15 °C. F1* was in excess and the samples were analyzed by denaturing PAGE. FAM fluorescence was detected by a gel imager. The experiments were repeated three times, and the data were fitted as described in the text. The solid curves represent the curve fits. k_{obs} was estimated to be 0.338 min^{-1} (4 °C) and 0.882 min^{-1} (15 °C). These values were used to calculate the estimated k_{obs} at 42 °C (7.0 min^{-1}) using the Arrhenius equation.

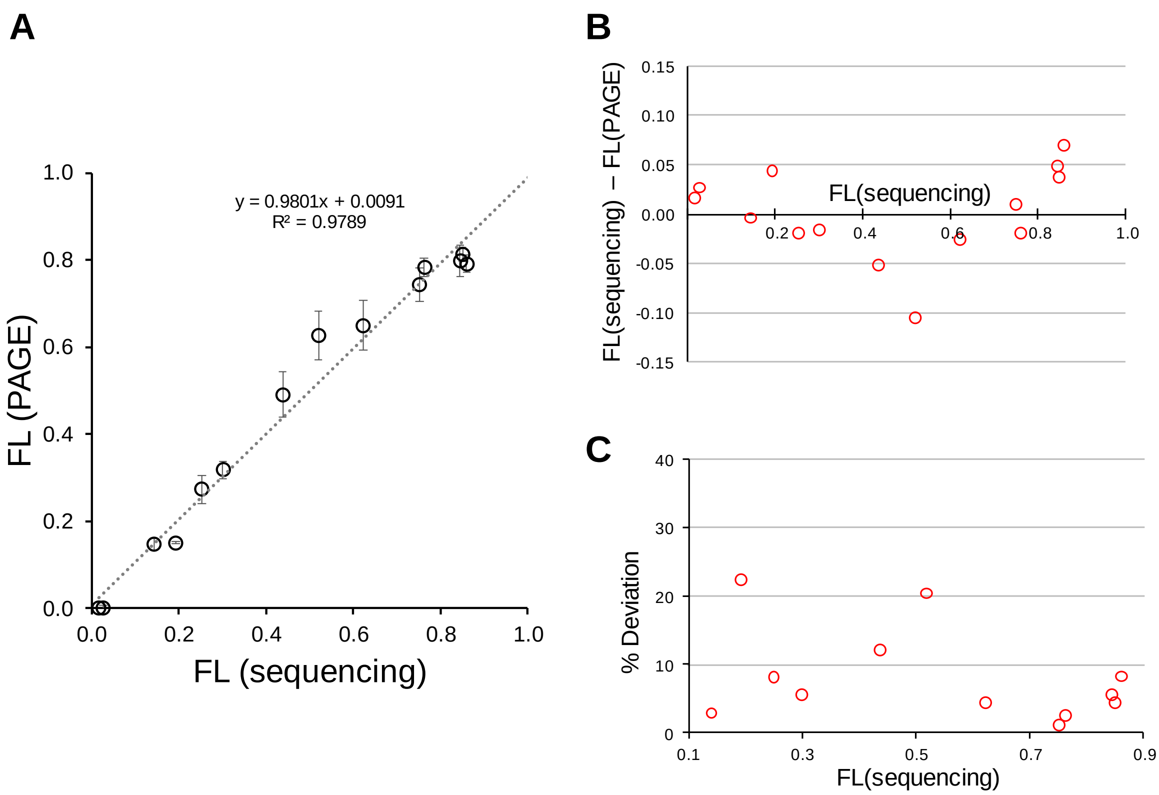

Figure S6. Correlation of ligase reactivities (FL) obtained from sequencing and individual PAGE assays. (**A**) minF1 and 13 variants were assayed. The ribozymes were ligated with fluorescently labeled substrate (FAM-F1*subA) with the ribozymes in excess, and the FAM-labeled substrate and the ligation products were separated by PAGE. PAGE analysis was performed at least twice per ribozyme variant, and the error bars indicate the minimum and the maximum values obtained. (**B**) Deviations of FL(sequencing) and FL(PAGE) data. Data shown in (**A**) were replotted for FL(sequencing)-FL(PAGE) vs FL(sequencing). Most data points fell within ~±0.05. (**C**) The absolute deviations in (**B**) expressed as a percentage of FL(sequencing). Most data points fell within ~10%. Two data points with no detectable product (FL=0) by PAGE were not included.

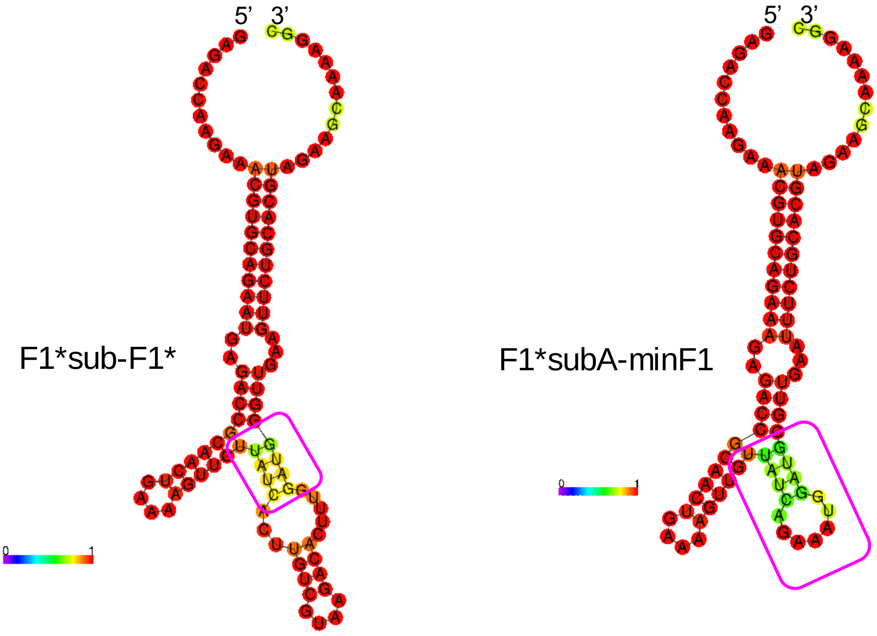

Figure S7. Predicted secondary structures of the ligated products of F1* (left) and minF1 (right) by the *RNAfold* web server.

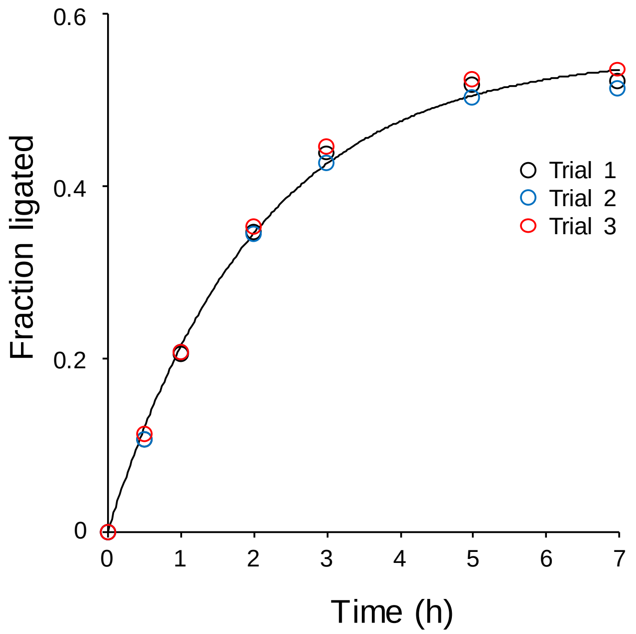

Figure S8. Ligation reaction of 4d394 and FAM-F1*subA. 4d394 was in excess and the samples were analyzed by denaturing PAGE. FAM fluorescence was detected by a gel imager. The experiment was repeated three times, and the data were fitted as described in the text. The solid curve represents the curve fit.

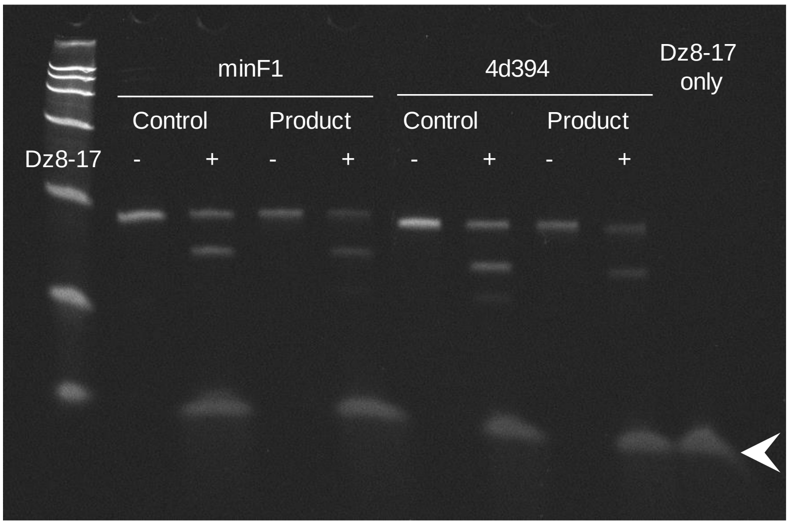

Figure S9. Deoxyribozyme (DNAzyme) cleavage of the ligation products to probe the regiospecificity of the ligation reaction. Ligation products of minF1 and 4d394 with F1*subA were purified and cleaved by excess deoxyribozyme 8-17 designed to target the ligation junction. Control ligation products were obtained by *in vitro* transcription of the expected ligation products. After the reaction, the deoxyribozyme was digested by TURBO DNase. The arrowhead indicates the band attributed to digested DNA.

Table S1. Oligonucleotides used in this study

Name	Sequence (5’ to 3’)
Ligase-lib-f	CCTAATACGACTCACTATAGAGACCGCA
Ligase-lib-r	GCCTTTTGCTTCTACGTGCAGAA
R1-504+2nt-F1*lig	ACACGACGCTCTTCCGATCTTATCCTCTGCCTTTTGCTTCTACGTGCA
R1-503-F1*lig	ACACGACGCTCTTCCGATCTAGAGTAGACAGCCTTTTGCTTCTACGTGCA
R2-F1*lig	GAGTTCAGACGTGTGCTCTTCCGATCTGAGACCGCAACTGAAAAGTTG
R1-f	ACACGACGCTCTTCCGATCT
Adapter-T2-new	AATGATACGGCGACCACCGAGATCTACACACACTCTTTCCCTACACGACGCTCTTCCGATC
B2-CGAGTAAT	CAAGCAGAAGACGGCATACGAGATCGAGTAATGTGACTGGAGTTCAGACGTGTGCTCTTCC
Dz8-17	TTTCAGTTGCGGTCTTCCGAGCCGGACGATTCTGCACGTTTCTTGG

Table S2. Relative activities of specially designed F1* mutants

Mutant code	Sequence (mutations are shown in lower case)	Description	RA
WT	GAGACCGCAACUGAAAAGUUGUUAUCACUUGUCGUAAGACACUUUGGAUGGGUUGAAGUUCUGCACGUAGAAGCAAAAGGC	"Wild-type" F1*	1.00
s1	GAGACCGCAACUGAAAAGUUGcUAUCACUUGUCGUAAGACACUUUGGAUGGGUUGAAGUUCUGCACGUAGAAGCAAAAGGC	GU to GC mutation at ribozyme base	0.99
s2	GAGACCGCAACUGAAAAGUUGUUAUCACUgcGUAAgcCUUUGGAUGGGUUGAAGUUCUGCACGUAGAAGCAAAAGGC	4-base deletion in the P5 stem	0.99
s3	GAGACCGCAACUGAAAAGUUGUUAUCACUggggGUAAccccCUUUGGAUGGGUUGAAGUUCUGCACGUAGAAGCAAAAGGC	Stronger P5 stem	1.04
s4	GAGACCGCAACUGAAAAGUUGUUAUCACUgggcGUAAgcccCUUUGGAUGGGUUGAAGUUCUGCACGUAGAAGCAAAAGGC	Stronger P5 stem	1.02
s5	GAGACCGCAACUGAAAAGUUGUUAUCACUggcgGUAAcgccCUUUGGAUGGGUUGAAGUUCUGCACGUAGAAGCAAAAGGC	Stronger P5 stem	1.01
s6	GAGACCGCAACUGAAAAGUUGUUAUCACUgcggGUAAccgcCUUUGGAUGGGUUGAAGUUCUGCACGUAGAAGCAAAAGGC	Stronger P5 stem	1.00
s7	GAGACCGCAACUGAAAAGUUGUUAUCACUggccGUAAggccCUUUGGAUGGGUUGAAGUUCUGCACGUAGAAGCAAAAGGC	Stronger P5 stem	0.97
s8	GAGACCGCAACUGAAAAGUUGUUAUCACUgcgcGUAAgcgcCUUUGGAUGGGUUGAAGUUCUGCACGUAGAAGCAAAAGGC	Stronger P5 stem	1.03
s9	GAGACCGCAACUGAAAAGUUGUUAUCACUgccgGUAAcggcCUUUGGAUGGGUUGAAGUUCUGCACGUAGAAGCAAAAGGC	Stronger P5 stem	0.96
s10	GAGACCGCAACUGAAAAGUUGUUAUCACUgcccGUAAgggcCUUUGGAUGGGUUGAAGUUCUGCACGUAGAAGCAAAAGGC	Stronger P5 stem	1.02
s11	GAGACCGCAACUGAAAAGUUGUUAUCACUcgggGUAAcccgCUUUGGAUGGGUUGAAGUUCUGCACGUAGAAGCAAAAGGC	Stronger P5 stem	1.01
s12	GAGACCGCAACUGAAAAGUUGUUAUCACUcggcGUAAgccgCUUUGGAUGGGUUGAAGUUCUGCACGUAGAAGCAAAAGGC	Stronger P5 stem	1.03
s13	GAGACCGCAACUGAAAAGUUGUUAUCACUcgcgGUAAcgcgCUUUGGAUGGGUUGAAGUUCUGCACGUAGAAGCAAAAGGC	Stronger P5 stem	0.99
s14	GAGACCGCAACUGAAAAGUUGUUAUCACUccggGUAAccggCUUUGGAUGGGUUGAAGUUCUGCACGUAGAAGCAAAAGGC	Stronger P5 stem	1.02
s15	GAGACCGCAACUGAAAAGUUGUUAUCACUcgccGUAAggcgCUUUGGAUGGGUUGAAGUUCUGCACGUAGAAGCAAAAGGC	Stronger P5 stem	0.99
s16	GAGACCGCAACUGAAAAGUUGUUAUCACUccgcGUAAgcggCUUUGGAUGGGUUGAAGUUCUGCACGUAGAAGCAAAAGGC	Stronger P5 stem	1.04
s17	GAGACCGCAACUGAAAAGUUGUUAUCACUcccgGUAAcgggCUUUGGAUGGGUUGAAGUUCUGCACGUAGAAGCAAAAGGC	Stronger P5 stem	1.08
s18	GAGACCGCAACUGAAAAGUUGUUAUCACUccccGUAAggggCUUUGGAUGGGUUGAAGUUCUGCACGUAGAAGCAAAAGGC	Stronger P5 stem	1.05
s19	GAGACCGCAACUGAAAAGUUGUUAUCACUUGUCGUAAGACACUUUGGAUGaGUUGAAGUUCUGCACGUAGAAGCAAAAGGC	Mutation in the P2 stem	0.96
s20	GAGACCGCAACUGAAAAGUUGUUAUCACUUGUCGUAAGACACUUUGGAUGcGUUGAAGUUCUGCACGUAGAAGCAAAAGGC	Mutation in the P2 stem	0.98
s21	GAGACCGCAACUGAAAAGUUGUUAUCACUUGUCGUAAGACACUUUGGAUGuGUUGAAGUUCUGCACGUAGAAGCAAAAGGC	Mutation in the P2 stem	0.96
s22	GAGACCGCAACUGAAAAGUUGUUAUCACUUGUCGUAAGACACUUUGGAUGGaUUGAAGUUCUGCACGUAGAAGCAAAAGGC	Mutation in the P2 stem	1.00
s23	GAGACCGCAACUGAAAAGUUGUUAUCACUUGUCGUAAGACACUUUGGAUGGcUUGAAGUUCUGCACGUAGAAGCAAAAGGC	Mutation in the P2 stem	0.88
s24	GAGACCGCAACUGAAAAGUUGUUAUCACUUGUCGUAAGACACUUUGGAUGGuUUGAAGUUCUGCACGUAGAAGCAAAAGGC	Mutation in the P2 stem	0.84
s25	GAGACCGCAACUGAAAAGUUGUUAUCACUUGUCGUAAGACACUUUGGAUGGGaUGAAGUUCUGCACGUAGAAGCAAAAGGC	Mutation in the P2 stem	1.01
s26	GAGACCGCAACUGAAAAGUUGUUAUCACUUGUCGUAAGACACUUUGGAUGGGcUGAAGUUCUGCACGUAGAAGCAAAAGGC	Mutation in the P2 stem	1.03
s27	GAGACCGCAACUGAAAAGUUGUUAUCACUUGUCGUAAGACACUUUGGAUGGGgUGAAGUUCUGCACGUAGAAGCAAAAGGC	Mutation in the P2 stem	1.01
s28	GAGACCGCAACUGAAAAGUUGUUAUCACUUGUCGUAAGACACUUUGGAUGGGUaGAAGUUCUGCACGUAGAAGCAAAAGGC	Mutation in the P2 stem	1.00
s29	GAGACCGCAACUGAAAAGUUGUUAUCACUUGUCGUAAGACACUUUGGAUGGGUcGAAGUUCUGCACGUAGAAGCAAAAGGC	Mutation in the P2 stem	1.02
s30	GAGACCGCAACUGAAAAGUUGUUAUCACUUGUCGUAAGACACUUUGGAUGGGUgGAAGUUCUGCACGUAGAAGCAAAAGGC	Mutation in the P2 stem	0.98
s31	GAGACCGCAACUGAAAAGUUGUUAUCACUUGUCGUAAGACACUUUGGAUGGGUUGAAaUUCUGCACGUAGAAGCAAAAGGC	GU to AU mutation at the ligation junction	1.03
s32	GAGACCGCAACUGAAAAGUUGUUAUCACUUGUCGUAAGACACUUUGGAUGGGUUGAAcUUCUGCACGUAGAAGCAAAAGGC	Mismatch at the ligation junction	0.93
s33	GAGACCGCAACUGAAAAGUUGUUAUCACUUGUCGUAAGACACUUUGGAUGGGUUGAAuUUCUGCACGUAGAAGCAAAAGGC	Mismatch at the ligation junction	1.01
s34	GAGACCGCAACUGAAAAGUUGUgcUCgaUUGUCGUAAGACAgUUUGaAUGGGUUGAAGUUCUGCACGUAGAAGCAAAAGGC	R3C core sequence	0.98
s35	GAGACCGCAACUGAAAAGUUGUgUAUCACUUGUCGUAAGACACUUUGGAUGGUUGAAGUUCUGCACGUAGAAGCAAAAGGC	Reduce P2 to 3-bp	0.29
s36	GAGACCGCAACUGAAAAGUUGUggUAUCACUUGUCGUAAGACACUUUGGAUGUUGAAGUUCUGCACGUAGAAGCAAAAGGC	Reduce P2 to 2-bp	0.00
s37	GAGACCGCAACUGAAAAGUUGUgguUAUCACUUGUCGUAAGACACUUUGGAUGUGAAGUUCUGCACGUAGAAGCAAAAGGC	Reduce P2 to 1-bp	0.00
s38	GAGACCGCAACUGAAAAGUUGUAUCACUUGUCGUAAGACACUUUGGAUGuGGUUGAAGUUCUGCACGUAGAAGCAAAAGGC	Increase P2 to 5-bp	0.84

Table S3. Relative activities of minF1 mutants in the G20-A22 bulge

Bulge sequence	RA	Bulge sequence	RA	Bulge sequence	RA	Bulge sequence	RA
GAA (WT)	1.00	3-nt bulge, double mutants		3-nt bulge, triple mutants		2-nt bulge	
3-nt bulge, single mutants		acA	0.01	acc	0.00	AA	0.18
aAA	0.48	agA	0.08	acg	0.00	AC	0.00
cAA	0.02	auA	0.01	acu	0.00	AG	0.01
uAA	0.15	ccA	0.01	agc	0.00	AU	0.01
GcA	0.15	cgA	0.10	agg	0.00	CA	0.02
GgA	0.11	cuA	0.00	agu	0.00	CC	0.00
GuA	0.17	ucA	0.00	auc	0.00	CG	0.00
GAc	0.85	ugA	0.15	aug	0.00	CU	0.00
GAg	1.04	uuA	0.00	auu	0.00	GA	0.84
GAu	1.04	aAc	0.03	ccc	0.00	GC	0.01
		aAg	0.09	ccg	0.00	GG	0.01
		aAu	0.10	ccu	0.00	GU	0.01
		cAc	0.00	cgc	0.00	UA	0.06
		cAg	0.01	cgg	0.01	UC	0.00
		cAu	0.01	cgu	0.00	UG	0.00
		uAc	0.01	cuc	0.00	UU	0.00
		uAg	0.02	cug	0.00	1-nt bulge	
		uAu	0.03	cuu	0.00	A	0.01
		Gcc	0.00	ucc	0.00	C	0.00
		Gcg	0.01	ucg	0.00	G	0.00
		Gcu	0.01	ucu	0.00	U	0.00
		Ggc	0.01	ugc	0.00	No bulge	
		Ggg	0.00	ugg	0.00	-	0.00
		Ggu	0.01	ugu	0.00		
		Guc	0.00	uuc	0.00		
		Gug	0.01	uug	0.00		
		Guu	0.01	uuu	0.00		

For the 3-nt bulge mutants, the mutations are indicated in lowercase.

References

1. Ikawa, Y., Tsuda, K., Matsumura, S. and Inoue, T. (2004) De novo synthesis and development of an RNA enzyme. *Proc. Natl. Acad. Sci. U.S.A.*, **101**, 13750-13755.

2. Robertson, M.P., Hesselberth, J.R. and Ellington, A.D. (2001) Optimization and optimality of a short ribozyme ligase that joins non-Watson-Crick base pairings. *RNA*, **7**, 513-523.
